# Population Density, Climate Variables and Poverty Synergistically Structure Spatial Risk in Urban Malaria in India

**DOI:** 10.1371/journal.pntd.0005155

**Published:** 2016-12-01

**Authors:** Mauricio Santos-Vega, Menno J Bouma, Vijay Kohli, Mercedes Pascual

**Affiliations:** 1 Department of Ecology and Evolution, University of Chicago, Chicago, United States of America; 2 London School of Hygiene and Tropical Medicine, University of London, United Kingdom; 3 Institute for Climate Sciences (IC3), University of Barcelona, Barcelona, Spain; 4 Ahmedabad Municipal Corporation, Ahmedabad, India; Imperial College London, UNITED KINGDOM

## Abstract

**Background:**

The world is rapidly becoming urban with the global population living in cities projected to double by 2050. This increase in urbanization poses new challenges for the spread and control of communicable diseases such as malaria. In particular, urban environments create highly heterogeneous socio-economic and environmental conditions that can affect the transmission of vector-borne diseases dependent on human water storage and waste water management. Interestingly India, as opposed to Africa, harbors a mosquito vector, *Anopheles stephensi*, which thrives in the man-made environments of cities and acts as the vector for both *Plasmodium vivax* and *Plasmodium falciparum*, making the malaria problem a truly urban phenomenon. Here we address the role and determinants of within-city spatial heterogeneity in the incidence patterns of vivax malaria, and then draw comparisons with results for falciparum malaria.

**Methodology/principal findings:**

Statistical analyses and a phenomenological transmission model are applied to an extensive spatio-temporal dataset on cases of *Plasmodium vivax* in the city of Ahmedabad (Gujarat, India) that spans 12 years monthly at the level of wards. A spatial pattern in malaria incidence is described that is largely stationary in time for this parasite. Malaria risk is then shown to be associated with socioeconomic indicators and environmental parameters, temperature and humidity. In a more dynamical perspective, an Inhomogeneous Markov Chain Model is used to predict vivax malaria risk. Models that account for climate factors, socioeconomic level and population size show the highest predictive skill. A comparison to the transmission dynamics of falciparum malaria reinforces the conclusion that the spatio-temporal patterns of risk are strongly driven by extrinsic factors.

**Conclusion/significance:**

Climate forcing and socio-economic heterogeneity act synergistically at local scales on the population dynamics of urban malaria in this city. The stationarity of malaria risk patterns provides a basis for more targeted intervention, such as vector control, based on transmission ‘hotspots’. This is especially relevant for *P*. *vivax*, a more resilient parasite than *P*. *falciparum*, due to its ability to relapse and the operational shortcomings of delivering a “radical cure”.

## Author Summary

Urbanization and environmental change are the main driving forces of ecological and social change around the globe, specifically in developing countries and for human health. Cities in developing countries exhibit rapid and unplanned urbanization which creates heterogeneous environmental and socio-economic conditions, which can in turn lead to different risks of infection. Here we address the role of urban spatial heterogeneity in infection risk by *Plasmodium vivax* with an extensive surveillance data set from Ahmedabad, India, spanning 12 years. This parasite is one of the four malaria species in humans, and is today the dominant cause of malaria in Indian cities. Our results show clear spatial structure in malaria incidence within the city, dependent on wealth, population density and the climate (temperature and humidity). Because this pattern of spatial risk is largely stationary in time, it can be incorporated in a prediction model and it identifies target ‘hotspots’ for intervention. Similar conclusions apply to the reported cases of *Plasmodium falciparum* which reinforces the importance of spatial heterogeneity in urban malaria more generally.

## Introduction

Addressing health problems associated with urban growth will be one of the major challenges of the 21st century, especially for the developing world [1]. City life is associated with significant variation in socioeconomic and environmental conditions of potential relevance to vector-borne diseases [2–4]. In particular, the pronounced and on-going increase in urban population [5, 6], combined with climate change and economic disparities could act synergistically on the transmission dynamics of malaria [7–9]. Although, Indian cities harbor both malaria parasites, *Plasmodium falciparum* and *Plasmodium vivax*, there is an increasing appreciation of the latter as a threat to global health [10], in particular in urban environments where *Plasmodium vivax* has become the most prevalent parasite. *Plasmodium vivax* has re-emerged in areas previously cleared of malaria, and has done so with higher mortality and morbidity than previously documented [11–13]. In Indian cities with seasonal transmission, the incidence of vivax malaria starts to rise earlier than that of falciparum malaria, and also earlier than transmission via the vector would allow. This earlier part of the vivax season is dominated and enabled by relapses. The parasite has the ability to delay the development of a fraction of the infectious load of sporozoites in the liver [14], which results in the relapse of the disease after the primary infection is cleared from the bloodstream [15]. The later part of the season is shared with *P*. *falciparum*, reflecting largely vector-borne transmission following the monsoon rains [10]. A better understanding of the spatial heterogeneity of malaria vivax risk (defined based on the wards’ incidence) within cities remains an open area of research in the population dynamics of the disease, of relevance to both prediction and intervention.

Historically, urbanization has led to economic and social transformations associated with profound improvements in sanitation and hygiene [16, 17]. For malaria, the process of urbanization is generally thought to reduce transmission, primarily because urban environments are largely unsuitable for malaria vectors due to a lack of breeding sites and the pollution of potential larval habitats [18]. Other explanations for reduced malaria risk include better access to health care services and an increased ratio of humans to mosquitoes [2]. There is concern however that areas with rapid unplanned urbanization and poor sanitation may not experience this marked decrease in malaria transmission [5].

The most common hypotheses for the persistence of malaria in cities include spatial variation in: 1) environmental conditions (relative humidity, temperature, precipitation), land use, and stored water, which create a favorable environment for Anopheles breeding in cities [7, 19, 20]; 2) socioeconomic factors (income, human movement, population density and the failure of local malaria intervention among others), which hamper the effectiveness of case management and the promotion of intermittent antimalarials [8, 21–24]; and 3) at more local scales, variation in mosquito behavior and ecology which can influence transmission intensity [22, 23]. Despite increased interest in the role of spatial heterogeneities in the population dynamics of vector-borne diseases [25–28], malaria models typically assume spatially homogeneous transmission and tend to aggregate temporal dynamics over space. In particular, they do not take into account how spatial variation in environmental, climatic and socio-economic conditions affect vector habitat, contact rates, host susceptibility and the effectiveness of control [27–29].

Importantly, these considerations are focused on Africa where endemic malaria remains a predominantly rural problem, because the main mosquito vectors are themselves rural, and in cities, largely peri-urban [2, 3, 5, 7, 8]. By contrast, the Indian subcontinent harbors a truly urban vector, *Anopheles stephensi*, particularly thrives in man-made environments, and breeds in various artificial containers within homes and in water collected in construction sites (catch basins, seepage canals, wells) [30], whereas its sister (sub) species (myorensis) is associated with rural areas. The existence of this particular vector within cities poses a unique challenge to the elimination of malaria in India. Cities can act as a reservoir for the persistence of the disease beyond their administrative limits, and prevent elimination despite considerable gains in the fight against rural transmission. But even beyond the Indian subcontinent, malaria can no longer be considered only a rural problem, given the increasing proportion of the world population living in cities and transmission of the disease in urban environments sometimes surpassing that of rural ones [2, 3, 9].

Here, we describe the spatial pattern of urban *Plasmodium vivax* risk within a large city of India, and investigate its association with socio-economic and environmental heterogeneity. By combining statistical analyses and adapting a probabilistic modeling framework previously proposed for cholera [31], we show that spatial heterogeneity in population size, environmental and economic factors, modulates malaria risk, and that the temporal effect of climate variables on malaria risk interacts with this spatial heterogeneity. These findings emphasize the importance of considering the interplay of climate forcing and socio-economic heterogeneity in the population dynamics of urban malaria in India. They also provide a basis for more targeted intervention, such as vector control, based on identifying transmission ‘hotspots’. Comparison to findings for *Plasmodium falciparum* reinforce the evidence for a role of spatial heterogeneity in the transmission of urban malaria in this region.

## Methods

### Data

**Fig 1:**
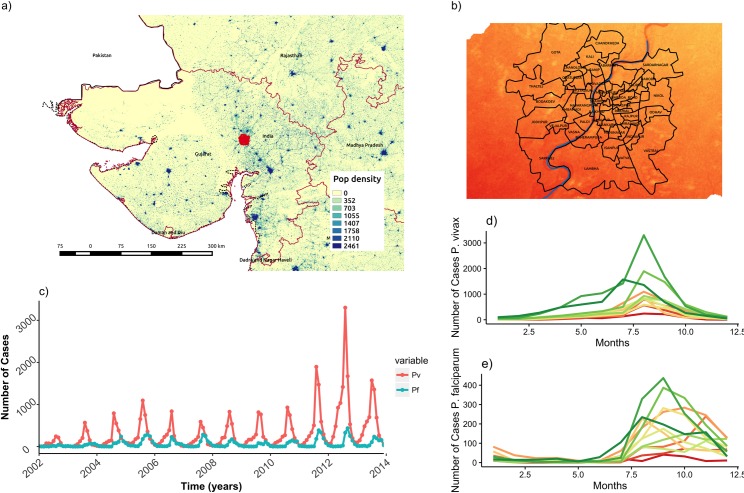
Study Area. Location of study area (A), and temporal patterns of incidence of *P*. *falciparum* (red solid) and *P*. *vivax* (blue dashed) (B, C). Boxplots are shown in B to illustrate seasonality, and time series are shown in C to illustrate interannual variation.

We take advantage of a highly disaggregated dataset of monthly malaria cases collected by the Municipal Corporation of the city of Ahmedabad, the capital of state of Gujarat in Northwest India (Fig 1A and 1B). In this largely semi-arid state where malaria is seasonally epidemic, Ahmedabad reports more than 1000 cases every year. The city presents ideal conditions to investigate malaria transmission dynamics in an urban environment, since it has experienced rapid development, unplanned urbanization and large population growth. It is also located on the banks of the Sabarmati River which creates environmental variation in this otherwise arid setting. The malaria data consist of monthly cases for the dominant parasite, *Plasmodium vivax*, over the last decade, confirmed through microscopy examination of blood slides from clinical (febrile) individuals self-presenting at the health facilities (Fig 1B and 1C). The resulting time series span a total of 12 years (from 2002 to 2014). Because some administrative units (known as wards) were subdivided into smaller wards in 2007, we aggregated the 64 units of the city into 59 wards, in order to maintain the same geographical subdivision through time. For comparison purposes, a similar data set for *Plasmodium falciparum* is also considered.

Apart from decadal census data, annual population data were provided by the Ahmedabad Municipal Corporation to approximate the population of each ward. Socio-economic data were obtained from the District Census Handbook of the concerned district for the year 2001 from the Directorate of Census Operations, Gujarat. Monthly time series (from 2002 to 2014) for mean temperature, mean rainfall and relative humidity at 8 am are those from the meteorological station of the city of Ahmedabad (and were provided by the Indian Institute of Technology in Gandhinagar).

### Spatial regularities in malaria transmission

In order to investigate the existence of a spatial pattern in malaria incidence within the city of Ahmedabad, we performed a series of statistical analyses to address whether malaria risk varied within the city and what factors explained this variation. First, we analyzed the spatial and temporal variation of malaria incidence and identified regions of high and low risk based on incidence. Second, we performed a series of statistical analyses on the role of socioeconomic and environmental factors in the spatial, and spatio-temporal patterns of malaria incidence. These analyses ranged from a simple t-test comparing socio-economic factors between the two regions of differential malaria risk, to time series models incorporating the autocorrelation in the data and the external drivers (including climatic ones), to a full spatio-temporal general linear mixed model with random effects. Third, based on these results a probabilistic dynamical model was formulated for malaria transmission at the ward level, and predictions of this model were evaluated at the city level.

To consider a measure of vivax malaria risk independent from interannual variation, we normalized malaria incidence for each ward in a given year by the total number of cases throughout the city. We then ranked these normalized values across wards to determine if high risk locations were consistently so over time. To examine the robustness of the patterns, we complemented the estimation of the intensity of infection with the Slide Positivity Rate (SPR) [32] S1 Fig 1. SPR, defined as the number of laboratory-confirmed malaria cases per 100 suspected cases examined, provides a rapid and inexpensive means of assessing the burden of malaria in the population that relies on health care facilities. SPR provides a better estimate of the true intensity of malaria than malaria incidence (reported cases divided by population), as it is more robust to spatial sampling errors/biases resulting from variation in the population size of the wards [32]. SPR is independent from incidence, although it can be prone to different biases (as the result for example of other febrile diseases which would increase the number of blood tests taken). Confirming that the patterns are consistent across these two measures of malaria intensity is important because the population denominator that is relevant for the reporting may vary in ways that are difficult to determine. Also, the local population size might be under- or overestimated by a census carried out only once every 10 years in India.

To further characterize spatial variation in risk we applied a 2k-means cluster algorithm to the incidence data at the wards level, and examined the existence of at least two regions differing in malaria risk. (We pre-determined two groups of wards to consider the hypothesis of different transmission intensity in the core and periphery of the city). Also, two groups allow us to consider the subdivision of wards into high and low risk regions. We hypothesized that differences in malaria risk in these two main areas are largely explained by demographic and socio-economic factors. To test if the two regions differed significantly in those variables, we first extracted socioeconomic indicators, including slum density (number of slums/ward area), unemployment (number of unemployed people), number of marginal workers, literacy, population below 6 years, total population, number of households, vulnerable and economically deprived communities, from the 2001 Ahmedabad census and calculated the density of slums per ward based on cartographic information on the slums’ distribution within the city S2 Fig. A t-test was applied to evaluate if these two regions differed significantly in these covariates.

We then addressed whether the temporal variation in malaria incidence responded differentially to climate variables (rainfall, temperature and humidity) across the two regions. To determine which predictors explain the temporal variation in vivax cases, we considered models with autoregressive terms to account for serial correlation in the data. The correlation structure in the malaria time series was assessed by inspecting the autocorrelation function ACF. Then we applied a generalized linear model (GLM) framework. Because observed count data, such as reported cases in infectious diseases, often exhibit significant over-dispersion [33], a negative binomial distribution of cases was used. Backwards model selection was based on the minimum Akaike Information Criterion (AIC) (S1 Table). Variables with coefficient significantly different from zero were selected. Spatial correlation was incorporated by assuming a conditional autoregressive (CAR) process in the random effect *v*_*i*_:

$$v_{i}v_{j}=N\left(\frac{\sum_{j}a_{ij}v_{j}}{\sum_{j}a_{ij}},\frac{\sigma_{v}^{2}}{\sum_{j}a_{ij}}\right)$$

where *σ*^2^ controls the strength of the local dependence, and *a*_*ij*_ are neighborhood weights for each ward based on distance to the river. We additionally compared the best model to a model with a different distribution (Zero Inflated Poisson) but this model was not significantly better (S2 Table). Finally, we considered the full spatio-temporal variation at the level of wards, by fitting a generalized linear mixed model including as covariates the effects of temperature and humidity, and a spatially-structured random effect weighted by the distance to the river (SSRE). Parameters and their distributions were estimated with Bayesian Markov Chain Monte Carlo (MCMC) parameter sampling implementation in WINBUGS.

### Probabilistic model for malaria dynamics

To model malaria risk within the city of Ahmedabad an inhomogeneous Markov chain model was used, following the theoretical framework developed for cholera by Reiner et al. 2012. In this approach the monthly malaria cases are categorized into discrete states of malaria incidence, which we chose as “low malaria”, “mild malaria,” and “high malaria”. The three discrete states partitioned the distribution of monthly incidence based on the 25^th^, lower than 75^th^ and above 75^th^ quantiles. Then, the model assigns baseline probabilities *P*_*i*,*j*_ to the transitions between these states in a defined time step as described by the following transition probability matrix P:

$$P_{i,j}=\left[\begin{array}{ccc} P_{1,1} & \left(1-P_{1,1}-P_{1,3}\right) & P_{1,3}\\ P_{2,1} & \left(1-P_{2,1}-P_{2,3}\right) & P_{2,3}\\ P_{3,1} & \left(1-P_{3,1}-P_{3,3}\right) & P_{3,3} \end{array}\right]$$

Where the baseline probabilities depend on the state of the system only in the previous time step. Modification of this basic matrix can introduce the effect of covariates, including the consideration of different regions such as the two groups of wards, identified in previous analyses. Thus, the resulting Markov chain model can be made inhomogeneous by allowing transition probabilities to depend on temporal and spatial environmental drivers; here seasonality, the state of the neighboring wards, temperature, Relative humidity and socioeconomic heterogeneity (summarized by the two groups of wards):

$$P_{i,j,k,t}=P_{i,j,d}\cdot Seas_{i,j,t,d}\cdot Neigh_{i,j,v,d}\cdot temp_{t,d}\cdot RH_{t,d}$$

Where *P*_*i*,*j*,*k*,*t*_ is the probability that ward k goes from state i to j from time t to time t+1. This probability is dependent on: (1) *P*_*i*,*j*,*d*_, the baseline transition probabilities of moving from state i to state j for a ward in risk region d; (2) a seasonal factor ${Seas}_{i,j,t,d}={\left(1+\beta_{i,j,d}\right)}^{{Se}_{\left(t,d\right)}}$, where the seasonality exponent *Se*_(*t*,*d*)_ is periodic over the 12 months of the year and each group d has its own seasonality; (3) a neighborhood effect ${Neigh}_{i,j,v,d}={\left(1+\propto_{i,j,d}\right)}_{t,d}^{v}$, where *v* corresponds to the malaria state (0,1,2) of the neighboring ward with the highest value. This function reduces to 1 when none of the neighboring wards have malaria reported. The parameters of these functions, *β*_*i*,*j*,*d*_ and ∝_*i*,*j*,*d*_ are estimated. Finally, the effects of temperature and humidity are included as sigmoidal functions, similar to the formulation for ENSO and its effect on cholera in Reiner et al 2012: ${temp/Rh}_{i,j,t,d}=\left(A_{d}tan(h_{d}\frac{{Temp/RH}_{t-\gamma }}{\left(2M_{d}\right)})tan(\frac{h_{d}}{2})\right)$ where ${Temp/{Rh}_{t}}_{i,j,d}\;is$ the temperature or humidity with temporal lag *γ*, in this case 2 months for the humidity and 1 month for the temperature, A is the maximal amplitude, M controls the scale (normalization by the maximum values allowed for the climate factors), and h is shape factor varying between 0 and ~ π to go from a linear to a nonlinear effect (S3 Fig).

We considered different models obtained by including or neglecting the effect of a subset of the following factors: temperature, relative humidity, the state of the neighboring wards and the two different risk regions. We compared each of the models to a null model) employing a likelihood ratio test. The most complex model has 78 parameters (S3 Table). Under Markovian assumptions, the transitions for the different time steps (months) are independent from each other [31, 34], which allows us to explicitly write a likelihood. The constraint that each transition probability must be between 0 and 1 was imposed by a barrier method (i.e. by setting the probability to 0 whenever its estimated value falls outside these limits) [31]. Each model was fitted by maximizing the likelihood with a Nelder-Meade simplex algorithm, which allows for the incorporation of such constraints.

Finally, to assess prediction performance, a cross-validation approach was implemented by sequentially removing the epidemic months (August-November) that follow the monsoon in a given year, refitting the model to the remaining data, and simulating it four months ahead starting from August to predict the course of the seasonal outbreak for the omitted period. Forecasting accuracy was estimated by computing the likelihood of the observed state. To that end, we inferred the probability distribution of the predicted state by performing 5000 independent simulations. This procedure is then sequentially repeated removing, one at a time, all the epidemic seasons available. To quantify the accuracy of our predictions, we calculate the percentage mean absolute error in our predictions, as well as a second quantity more practical and possibly relevant to public health, based on the definition of a ‘large’ outbreak. We defined such as event as one where the peak of the epidemic at the whole city level exceeds the 75% quantile of the distribution of this quantity. We quantify the fraction of times the model correctly predicts the observed malaria incidence state (above the 75% quantile). Then, to examine and illustrate the importance of the climate covariates to the predictions, we simulated the model using different combinations of humidity and temperature ‘data’. In particular, predictions for the epidemic months in the anomalous, low incidence years, were obtained using: 1) monthly observed temperature and average humidity 2) monthly average temperature and observed humidity 3) monthly average humidity and average temperature. Monthly averages were computed based on the mean of all previous years for a given month. We also examined the performance of the model by obtaining a one-step ahead prediction, where we removed 1 month of data at a time for all the wards (S4 Fig).

## Results

**Fig 2:**
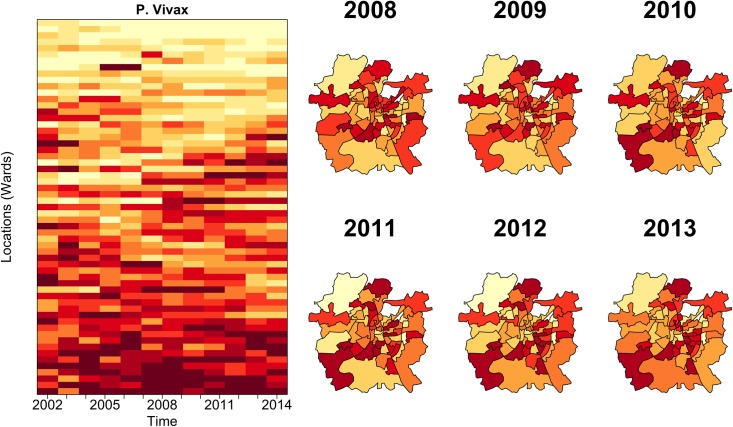
Analysis of spatio-temporal patterns of malaria vivax incidence in the city of Ahmedabad. The panels show the distribution of the cases normalized by population, with the intensity of the color (from low yellows to high reds) corresponding to the ranking of incidence. There is striking consistency from one year to the next in the places exhibiting the highest burden of the disease. Some of this regularity also extends to the two parasites. See S2 Fig, for comparison with the patterns obtained with SPR (Slide Positivity Rate) as an alternative measure of malaria intensity see S1 Fig.

We initially addressed whether the spatial distribution of normalized *Plasmodium vivax* risk is heterogeneous throughout the city, and how the ranking of risk varies in time. The top left panel of Fig 2 shows that several locations systematically rank high or low based on their *P*. *vivax* incidence through time. The top right panel in this figure reveals a stable spatial regularity of the locations with the highest malaria burden through time. This remarkable temporal stability of the spatial pattern suggests the existence of strong underlying determinants that are largely stationary at the temporal scales of malaria transmission within the city.

**Fig 3:**
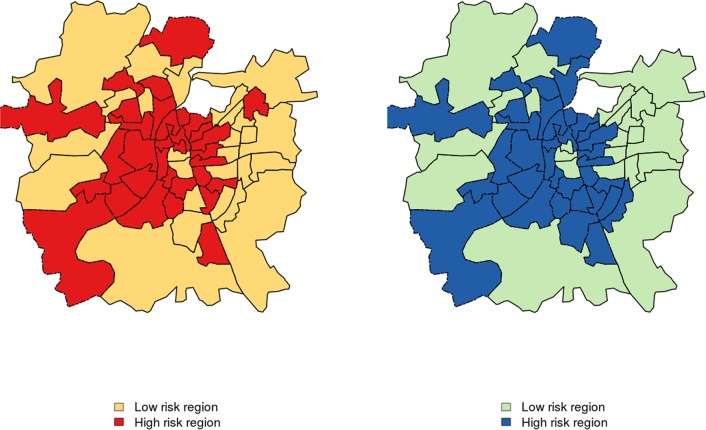
Map depicting the two groups of wards (administrative units). Map depicting the two groups of wards (administrative units), with high and low malaria risk respectively, *P*. *vivax* (left) and *P*. *falciparum* (right). There are significant differences in the annual malaria incidence between the two regions (p<0.001), for both parasites.

The spatial pattern observed in Fig 2 also indicates the existence of two distinct malaria risk regions within Ahmedabad: one comprised of the wards close to the Sabarmati River and the core of the city (region 1), and the other, of those in the newer urban periphery (region 2). Fig 3 shows the results of the cluster analysis using the rankings data, demonstrating that the group of wards that are close to the river and in the inner part of the city (referred to as high risk region hereafter) have a different malaria risk than those in the in the periphery (low risk region). *Plasmodium vivax* cases in the region defined as high risk are significantly (p<0.001) higher than those in the region defined as low risk, whereas the seasonal pattern remains the same for both areas. This results hold for both parasites S5 Fig.

We then asked whether the differences in malaria risk between the low and high malaria risk regions are associated with differences in population density (number of slums, population size, number of households) or economic level (income, unemployment, literacy). Table 1 summarizes the results from statistical comparisons (t-test) between the high and low risk regions (for *P*. *vivax*) based on socioeconomic indicators from the 2001 census. We found that the high risk region has significantly higher unemployment, slum density, total population and number of households. In addition, we did not find significant differences in the mean area of the wards. The differences in slum density, unemployment, literacy, economically disadvantaged people and vulnerable communities (both Scheduled Castes, SC, and Scheduled Tribes, ST, the official designations given to two different groups of historically disadvantaged people) are pronounced, with the high risk region encompassing for example a density of slums that is at least 1.2 higher than that of the low risk region (Table 1).

**Table 1:** Statistical analysis of differences between the two regions for *P*. *vivax*, based on the socioeconomic information of the 2001 census.

*variable*	*statistic*.*t*	*parameter*.*df*	*p*.*value*	*log(mean of High risk)*	*log(mean of low risk)*
*Slum density*	1.834	54.99	0.042	3.261	2.747
*Unemployment*	3.059	29.21	0.004	10.82	8.828
*Marginal workers*	3.197	30.56	0.003	6.967	5.592
*Literacy*	3.217	29.18	0.003	10.884	8.566
*pop_below_6years*	3.275	29.43	0.002	9.095	7.120
*Total population*	3.237	29.18	0.003	11.20	8.808
*Area*	0.336	45.51	0.737	1.379	1.438
*Number of Households*	3.178	29.29	0.003	9.565	7.544
*Vulnerable communities*	3.210	40.75	0.002	6.207	4.825
*Economically deprive communities*	3.934	36.14	0.000	8.841	6.682

Moreover, the temporal variation in malaria incidence between the two regions could be influenced differentially by the environmental covariates. Table 2 show the results of the stepwise multiple linear regression between *Plasmodium vivax* cases and climate covariates (temperature, humidity and rainfall) and S6 Fig show the residuals of our best model for each region. The temporal variation in the low risk region responds predominantly to changes in temperature and humidity, whereas that of the high risk region is explained predominantly by humidity, although both parameters are seasonally associated with rainfall. Humidity would act in both the temporal and spatial dimensions, as the water table and associated ground moisture should be higher in the high risk region closer to the river, and this parameter is known to affect survival of the adult mosquitoes [35]. Thus, increased humidity (through changes in the water table) can lead to an earlier onset of the *Plasmodium vivax* season and to higher incidence S7 Fig. Finally, results for the general linear mixed model show that the best model includes the effects of temperature and humidity, and the random spatial variation in the data weighted by the distance to the river (S4 Table and S5 Table).

**Table 2:** Results of the best model for monthly cases as a function of environmental covariates for *Plasmodium vivax*.

Low risk region						
	Estimate	Std. Error	z value	Pr(>|z|)	2.50%	97.50%
ar1	0.63891	0.064	9.917	0	0.448	0.718
intercept	-1.257	2.126	-0.591	0.554	-13.702	9.159
temp	0.140	0.069	2.006	0.044	-0.035	0.725
RH	0.034	0.025	1.329	0.0183	0.027	0.258
High risk region						
	Estimate	Std. Error	z value	Pr(>|z|)	2.50%	97.50%
ar1	0.5835	0.0689	8.4637	0.0000	0.4483	0.7186
intercept	-2.2716	5.8323	-0.3895	0.6969	-13.7027	9.1594
RH	0.1775	0.0235	3.2948	0.0010	0.1332	0.3952

Interestingly, *Plasmodium falciparum* also exhibits stable regularities in risk levels within the city S8 Fig. We find the existence of two different regions with contrasting incidence, largely consistent with those for *Plasmodium vivax* Fig 3, and a significant difference in socioeconomic level and environmental conditions for these two regions (S6 Table and S7 Table).

For a more dynamical perspective, we used an inhomogeneous Markov chain model that incorporates the effect of spatial and temporal variation on malaria risk. Results are also consistent for both parasites. Specifically, the comparison of the different models analyzed (S3 Table and S8 Table), shows that the best model is the one that accounts for the effects of seasonality, neighbors, temperature, humidity, and includes the two different risk regions identified above (model 5 Table 3). To test the significance of the individual components of each model (alternative hypothesis) against a model including seasonality only (model number 1, null hypothesis) we employed a likelihood ratio test. Improvements in likelihood for models 3 to 8 are significant, and so are the effects of seasonality, temperature, neighbors and the two regions (p<0.05). Improvements in likelihood for model 2 (seasonal effect + neighborhood effect) are not significant at p = 0.05. Moreover, models that account for the effect of spatial heterogeneity, represented by including the two risk regions, tend to perform better than those that do not incorporate this effect (S3 Table and S8 Table).

**Table 3:** Likelihood comparison of the models showing the covariates included in each model. The best likelihood is for the model that incorporates seasonality, temperature, two regions and neighbors. The last column shows the result of a likelihood ratio test between the null model (model 1) and each of the other models.

*models*	*seasonality*	*Neighbors*	*Temperature*	*RH*	*Regions*	*Log lik*	*DF*	*AIC*	*LRT*
*Model 5*	+	+	+	+	+	-4821.559	78	9799.119	*
*Model 6*	+	+	+		+	-4903.554	72	9951.107	*
*Model 7*	+	+			+	-5670.935	66	11473.87	*
*Model 4*	+	+	+	+		-5894.961	39	11867.92	*
*Model 8*	+				+	-5932.35	54	11972.7	*
*Model 3*	+	+	+			-6573.846	36	13219.69	*
*Model 2*	+	+				-6648.966	33	13363.93	
*Model 1*	+					-6656.621	27	13367.24	--

**Fig 4:**
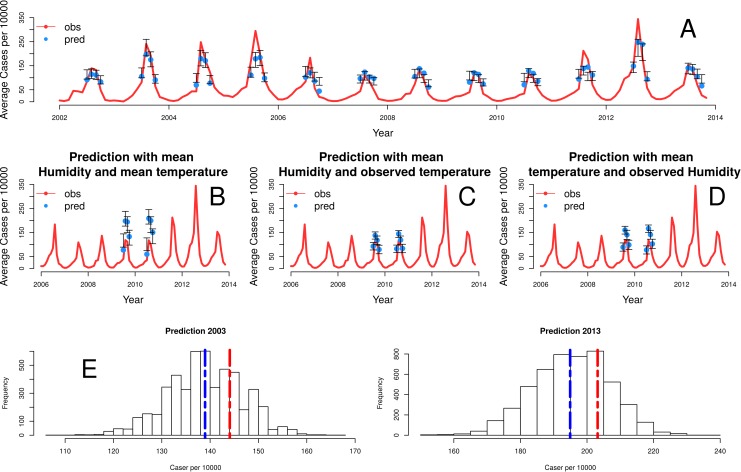
Comparison of observed and predicted cases with the best model. In (A), the red line corresponds to the average number of cases per 1000 for the 59 wards. The blue dots correspond to predictions given by the median of 5000 simulations, and the gray bars correspond to the 5th and 95th percentiles. In (B-D), simulations of the model predict the seasonal epidemics of 2009 and 2010 starting from the end of the monsoons (August) under modifications of the observed climate covariates. The different panels show the effect of fixing temperature and/or humidity at their mean monthly values, to remove their effect on the interannual variation of these anomalous years. When the interannual effect of both is removed (B), the model clearly over-estimates the cases. Individual effects are less pronounced (C, D) although predictions are also higher than observations. Our best model has a mean absolute error of 68% for predicting the peak of the epidemic in a year with a high number of cases (2013). Fig 4 (E, left), shows the distribution of model forecasts from 5000 runs for October 2003 based on October 2002 data. Although the mean prediction differs from the observation, almost all (~84%) model simulations resulted in large events for 2003. The figure on the right repeats this hindcast analysis for October 2013 (using data from 2012). Here, we find a reduced but still large (∼87%) probability of a large outbreak.

Finally, Fig 4 shows the predictions for the epidemic months for *Plasmodium vivax*, generated with the cross-validation procedure described above and the best model, which includes seasonality, temperature, the state of the neighboring wards, and spatial heterogeneity. The predicted rate of cases for the peak of the season is coherent with the observed rate. Further explorations of the model show the importance of temperature and humidity in the prediction of malaria risk Fig 4B. For example, the pronounced dip in the cases for years 2009 and 2010 is captured by the dependencies of the model on temperature and humidity, as those two years are dryer, warmer and less humid Fig 4C and 4D. Although our best model exhibits a tendency to under-predict the size of the peaks which we discuss below, it is able to capture the seasonality and to a reasonable extent, the interannual variation in the data. The anomalous decrease in the number of cases in 2009 and 2010 seasons can be explained by the variation in the climate covariates.

Because the model is stochastic and it considers discrete states (no malaria, low and high), we simulated repeatedly, and from these ensemble of simulations computed the mean number of cases in a given month for a given ward. Our simulations generate realizations of the stochastic process and therefore, configurations of the discrete states. To convert the discrete states to cases, we used the mean number of cases for each class. The red line corresponds to the wards mean observed cases of the city. The blue dots show the median of the simulated values and the blue shaded regions correspond to the 5th– 95th percentile range over 5000 simulations.

## Discussion

Most transmission models of vector-borne diseases tend to aggregate the data at large scales and treat transmission homogeneously in space [27–29]. However, at local scales spatial heterogeneity can significantly influence the risk of infection. In particular, urban environments can exhibit pronounced heterogeneity from rapid and unplanned urbanization. Spatial heterogeneity in the environmental conditions such as temperature and humidity or in socioeconomic level can affect mosquito ecology, such as habitat distribution, vector longevity, biting rate or host finding ability [36], and in factors related to human exposure and susceptibility respectively [8, 16, 37].

For *Plasmodium vivax* in Ahmedabad, we found defined heterogeneity in malaria risk that is slow-changing and therefore largely ‘stationary’ in time, relative to the characteristic temporal scales of the population dynamics of the disease. The presence of such stable pattern suggests strong and spatially-structured determinants of malaria risk. In particular, the existence of two main regions with different risk was shown to be associated with socioeconomic level. Higher risk is largely concentrated in the inner part of the city where socio-economic indicators reveal higher poverty on average (Fig 3 and Table 1). These results are consistent with the previously described negative associations between malaria and socioeconomic status [38–41]. Disease persistence decreases with increasing employment, literacy and income S1 Fig 9, with poor people more vulnerable to ineffective diagnosis and treatment for financial and cultural reasons, and less able to access antimalarial and anti-mosquito protection [42]. Consistent results for *Plasmodium falciparum* emphasize the driven nature of the patterns.

The two different risk regions within the city were also shown to exhibit differential temporal responses to climate forcing. This finding underscores the importance of humidity to malaria transmission, with a higher water table in the high risk region possibly increasing relative humidity and affecting vector ecology. The spread of malaria requires favorable conditions for the survival of both the mosquito and the parasite. Temperatures in the approximate range of 21°-32°C and a relative humidity of at least 60% are most conducive to transmission [38]. Malaria vectors need to live at least 8 days in order to transmit malaria, and higher humidity increases both survival rate and activity rate [43, 44]. These relationships explain why *Anopheles stephensi* is more active and prefers feeding during the night when relative humidity is higher. The low risk region should have lower humidity (ground moisture) given its distance from the Sabarmati river and other water bodies. It has been established that if the average monthly relative humidity (measured at 8 am) is below 60%, then the lifespan of the mosquito is too short leading to very low or no malaria transmission [4, 45, 46]. The effect of humidity is also evident in the estimated regression coefficients (Table 2), where the value corresponding to humidity is one order of magnitude higher for the high than the low risk region. This means that a typical annual change of 10% in humidity with the rest of the covariates kept the same for both regions, will result in an increase of 403 to 1030 additional malaria cases (or 15% to 39% relative to the mean) in the high-risk region, and 70 to 411 additional cases (or 7% to 33% relative to the mean) in the low risk region. Rainfall itself, which is closely related to both humidity and temperature, is not retained in the regression as a significant explanatory variable probably because of its collinear effects with the other climatic factors. Our results from the mixed model indicate however that while climate factors (temperature and humidity) play an important role in disease transmission, it is their combined effect with spatial heterogeneity in the risk weighted by the distance to the Sabarmati river that better explains the spatio-temporal variation in the malaria incidence. More mechanistic models informed by time series data on disease incidence and vector abundance, will provide further insight on the role and interplay of climate covariates through their effects on parasite mortality, as well as on vector breeding and longevity.

Social and economic elements such as the quality of housing can also favor the biological development of mosquitoes [47]. It is common in urban areas of India for water to be supplied irregularly; this leads to water storage within houses which creates multiple breeding sites for the mosquito in overhead tanks, cisterns and cement tanks [48]. Higher population density would result in higher water storage concentrations in close proximity to people. At the local scale spatial heterogeneity in urban malaria risk would follow from high density, especially where coexisting with poverty, as well as environmental microclimates from the proximity of water bodies.

Our dynamical and stochastic model captures the seasonal pattern and the main trends in the interannual variation of the malaria cases. Interestingly, most of the models that incorporate spatial structure, namely the two regions, perform better than the models that do not. This conclusion is consistent with the results for diarrheal diseases in Dhaka, where consideration of different parts of the city also improved model performance [31, 48]. Our best model identifies significant spatial effects at two different scales: (1) that of neighboring wards (p<0.01), where the probability of transitioning to a higher risk level depends of the level of the surrounding wards, and (2) that of the two regions (p<0.001) influenced socioeconomic and demographic level. Although our model under-predicts the size of outbreaks, this tendency is expected from the discretization of the cases into a small number of levels, which preserves the rankings of the cases over time but tends to reduce the magnitude of the peaks. Predictions in that scale can still be useful when evaluating the risk of an outbreak larger than a given selected threshold [31]. Although additional classes could be incorporated in the formulation of the model, this would rapidly increase the number of parameters.

The model is able to capture the interannual trends and in particular, the lower outbreaks of years like 2009 and 2010, based on the effect of climate covariates. These two years exhibit anomalous high temperature and low humidity (associated with low monsoon rainfall), only comparable to values in 2002, another year with low incidence S10 Fig. The predictions of Fig 4B, for temperature and humidity kept at monthly averages, show that the model over-predicts the number of cases Fig 4. Thus, the anomalous incidence is explained by the lower temperature and higher humidity. Fig 4C and 4D isolate the effect of each climate covariate on the prediction, showing a stronger effect of temperature on the temporal reduction of cases of those two years.

Besides prediction, the phenomenological modeling framework applied here is also useful to address the spatial scale at which to aggregate the data to consider process-based epidemiological models, in a way that balances reducing the noise with representing dominant spatial heterogeneity. Questions on the spatial scale of aggregation are specifically relevant to addressing climate forcing in the context of socio-economic heterogeneity. Given that pronounced changes in urbanization will co-occur with those in climate, these are fundamental questions for infectious disease dynamics within cities.

Although our approach is able to capture the interannual variation in the data and predict the peak of the epidemic, it could be improved in several directions. For example, one could incorporate in the model: (1) mobility fluxes derived from the spatial distribution of the population with movement models, to replace the near-neighbor effects on transition probabilities; (2) the explicit effect of population density on group-dependent parameters explicitly; (3) further analysis of the local effect of environmental heterogeneities such as river discharge and soil moisture on malaria incidence at higher resolution by increasing the number of groups in the model. Moreover, temporal changes of the city itself would be of interest, including changes in the local speed of urbanization, and their implications for mobility, population distribution and economic level.

The region has experienced strong malaria interventions in the last three decades reflected in the pronounced negative trends in the number of reported cases from the 1980s and 19990s to the 2000s. From 2000 onwards, malaria prevalence in the city of Ahmedabad has remained however fairly stationary. Although the spatio-temporal variation in intervention efforts could influence the results of our models. This is unlikely given that the interventions within the city are largely homogeneous in space (S11 Fig), with small differences between the high and low risk regions.

Our probabilistic model tends to underpredict the size of outbreaks. This bias results from the transformation of the incidence data into discrete malaria levels, which smooth’s out extreme events. The effect of this bias can be assessed and corrected by lowering the threshold probability (the proportion of simulations with large outbreaks) below 50% using ROC (Receiver-Operating-Curves) (Reiner et al., 2012). The number of discrete classes describing the malaria levels could also be increased or estimated to balance complexity and accuracy. At the limit, one could move to stochastic models that do not require such discretization, although their parameterization would present challenges related to model complexity.

A better understanding of urbanization and malaria is needed, since urban environments can contribute to the persistence of the disease and frustrate elimination efforts more broadly at a regional level, by creating a reservoir for the disease in cities that contributes to transmission in rural areas. In India, the earlier National Eradication Programs focused on rural areas, with urban malaria contributing to the resurgence of disease in the 1970s [48]. Urbanization and growing populations also exacerbate inequalities in access to water, and in so doing introduce variation in another fundamental but poorly understood environmental factor for vector-transmitted infections. In concert these two dimensions, environmental and socio-economic, define the relevant spatial scales at which to address transmission dynamics. They also define the existence of spatial 'hotspots' of high disease risk in urban environments, especially those of the developing world where economic inequality and variation in disease vulnerability can be pronounced. Identifying these hotspots for targeted intervention in urban environments, can contribute to the control of vector-transmitted diseases, and eventually to the elimination of malaria in India.

## Supporting Information

S1 Fig Analysis of spatio-temporal patterns of malaria slide positivity rate in the city of Ahmedabad. The panels show the distribution of SPR with the intensity of the color (from low yellows to high reds for *P*. *vivax* and from light yellow to blue for *P*. *falciparum*) corresponding to the ranking based on the intensity of the transmission.
(TIF)

S2 Fig Slum distribution in the city (left) and slum density calculation (right). The latter was generated by overlaying the slum distribution map with the wards map provided by the municipal corporation, and calculating the number of slums per ward divided by the ward area.
(TIF)

S3 Fig Functional forms of the humidity (bottom) and temperature (top) effects illustrating the flexibility of the formulation, with different values of parameter h leading to different shapes. (Here we show these functional forms for an arbitrary amplitude A and scale M, and different shape values: h = 0 (black line), h = 7/8pi (red line) and h = pi (green line).
(TIF)

S4 Fig Comparison of predictions and observations. The red line corresponds to the average monthly cases per 10000 for the 59 wards. The blue dotted line corresponds to one-month ahead predictions for the median of the 5000 simulations values, and the light brown shaded region corresponds to the interval between the 5^th^ and 95^th^ percentiles for these simulations.
(TIF)

S5 Fig Malaria seasonal pattern for the two risk regions. The top panels represent the seasonal pattern for *Plasmodium falciparum*, for the high risk region in the left and the low risk region in the right. The bottom panel shows the corresponding patterns for *Plasmodium vivax*.
(TIF)

S6 Fig The figure shows a time plot and ACF of the residuals for the fitted models. Although most of the autocorrelations fall within the confidence intervals, there is a small autocorrelation at lags of 11 and 12 months (seen in the significant spike of the ACF plot). This suggests that the model can be slightly improved by capturing the remaining seasonal variation.
(TIF)

S7 Fig The top panels show scatterplots of humidity vs cases for *P*. *vivax (left panels)* and *P*. *falciparum (right panels)*, and the bottom ones, the time series for the cases (in red for vivax and in blue for falciparum) together with those for humidity (in black). (TIF)

S8 Fig Analysis of spatio-temporal patterns of malaria incidence in the city of Ahmedabad (from low yellow to high blue). The panels show the distribution of the cases normalized by population, with the intensity of the color corresponding to the ranking of incidence.
(TIF)

S9 Fig Scatterplot of socioeconomic indicators (number of households, literacy and unemployment) and the number of cases of *P*. *falciparum* and *P*. *vivax* per ward. (TIF)

S10 Fig From left to right, scatterplots of the annual mean temperature, humidity and rainfall vs annual *Plasmodium vivax* incidence. (TIF)

S11 Fig The map depicts the mean number of containers treated with larvicide throughout the city. Red dots represent wards in the high risk regions, and yellow dots, those in the low risk region.
(TIF)

S1 Table Comparisons of nested models based on the likelihood ratio test (where asterisks indicate significance relative to the previous model). (DOCX)

S2 Table The table shows comparisons of the zero inflated Poisson and negative binomial models fitted to the data. (DOCX)

S3 Table Different parameterizations of the probabilistic model. The table shows the effects included in the model formulation and the corresponding total number of parameters.
(DOCX)

S4 Table Model comparisons highlight the best model which incorporates the random effects, as well as the effect of temperature and humidity. (DOCX)

S5 Table Estimated parameters for the best model which includes the effects of temperature relative and relative humidity, and the random effects, whose values are significantly different from zero. Confidence intervals (CI) from posterior distributions (from two chains that are well mixed and have converged).
(DOCX)

S6 Table Statistical analysis of differences between the two regions identified using the socioeconomic information of the 2001 census for *P*. *falciparum*. (DOCX)

S7 Table Results of the best negative binomial model selected by AIC for *Plasmodium falciparum* with climate covariates. This model includes an autoregressive term that accounts for serial autocorrelation, the effect of relative humidity in both regions, and the effect of temperature in the low risk region.
(DOCX)

S8 Table Likelihood comparison of the models showing the covariates included in each model. The best likelihood is obtained for the model that incorporates seasonality, temperature, two regions and the effect of neighbors. The last column shows the likelihood ratio test between the null model (model 1) and each of the other models.
(DOCX)
