# Supplementary figures and images for: Population Density, Climate Variables and Poverty Synergistically Structure Spatial Risk in Urban Malaria in India
Source: PLoS Negl Trop Dis. 2016 Dec 1;10(12):e0005155. doi: 10.1371/journal.pntd.0005155 (PMC5131912; doi:10.1371/journal.pntd.0005155)

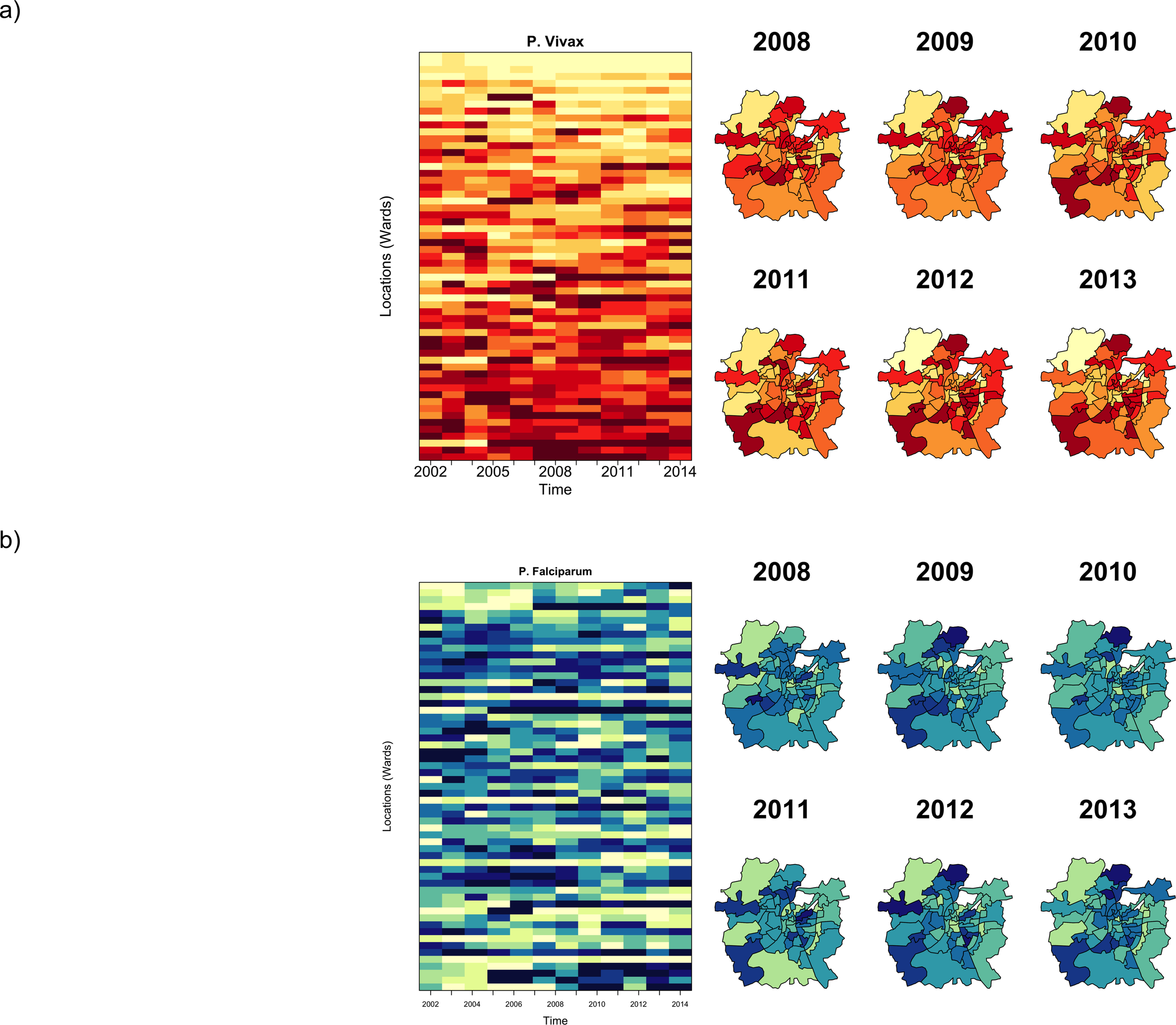

Supplement: S1 Fig — The panels show the distribution of SPR with the intensity of the color (from low yellows to high reds for P. vivax and from light yellow to blue for P. falciparum) corresponding to the ranking based on the intensity of the transmission. (TIF) [file pntd.0005155.s001.tif]

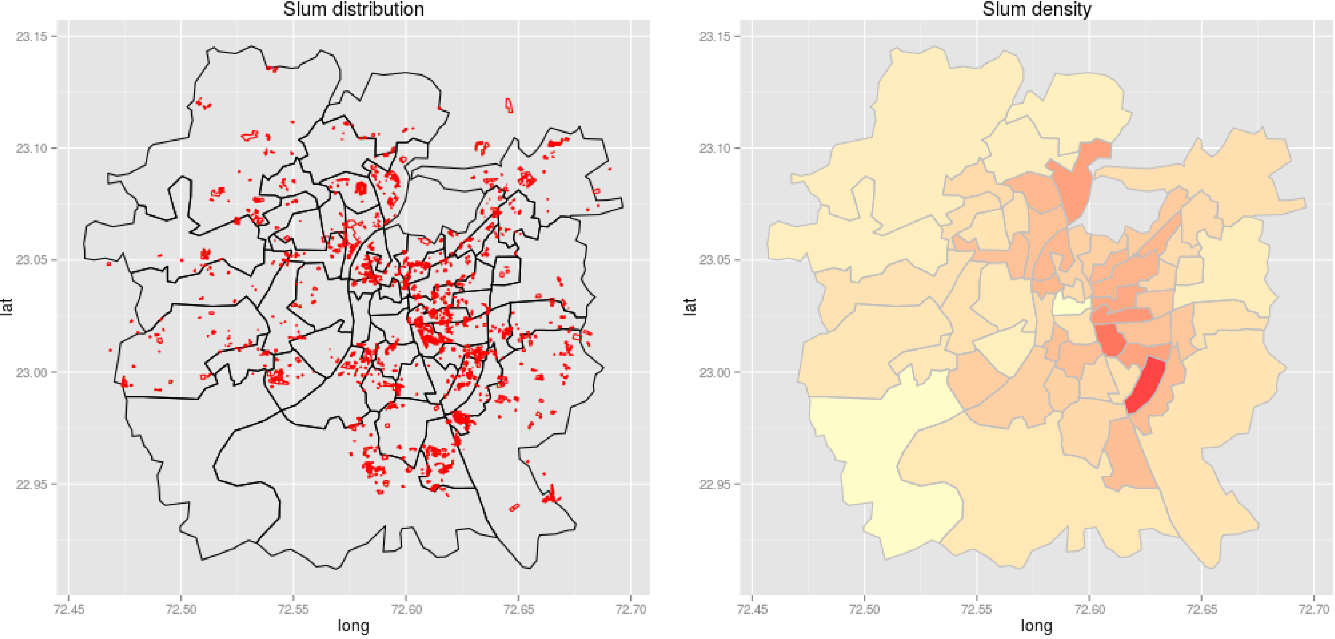

Supplement: S2 Fig — The latter was generated by overlaying the slum distribution map with the wards map provided by the municipal corporation, and calculating the number of slums per ward divided by the ward area. (TIF) [file pntd.0005155.s002.tif]

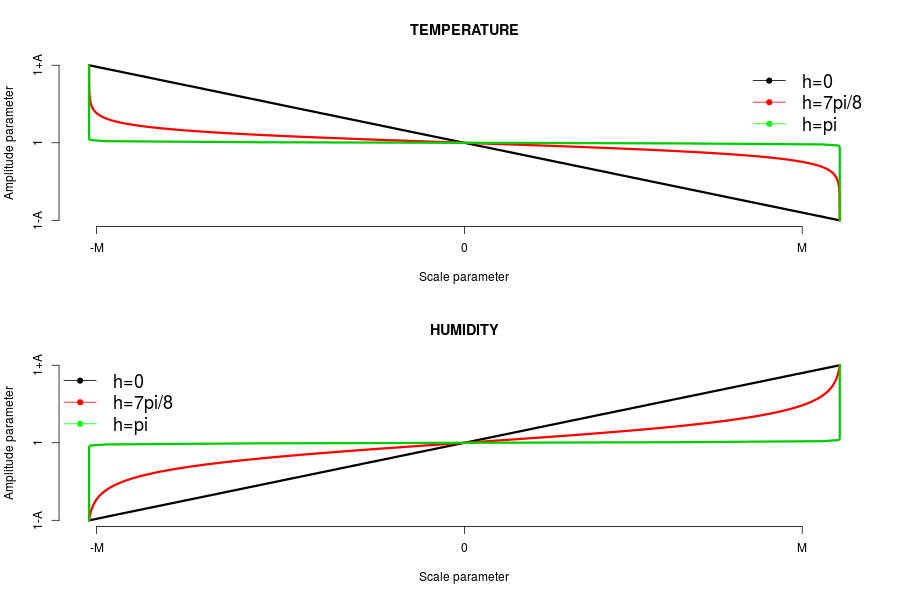

Supplement: S3 Fig — (Here we show these functional forms for an arbitrary amplitude A and scale M, and different shape values: h = 0 (black line), h = 7/8pi (red line) and h = pi (green line). (TIF) [file pntd.0005155.s003.tif]

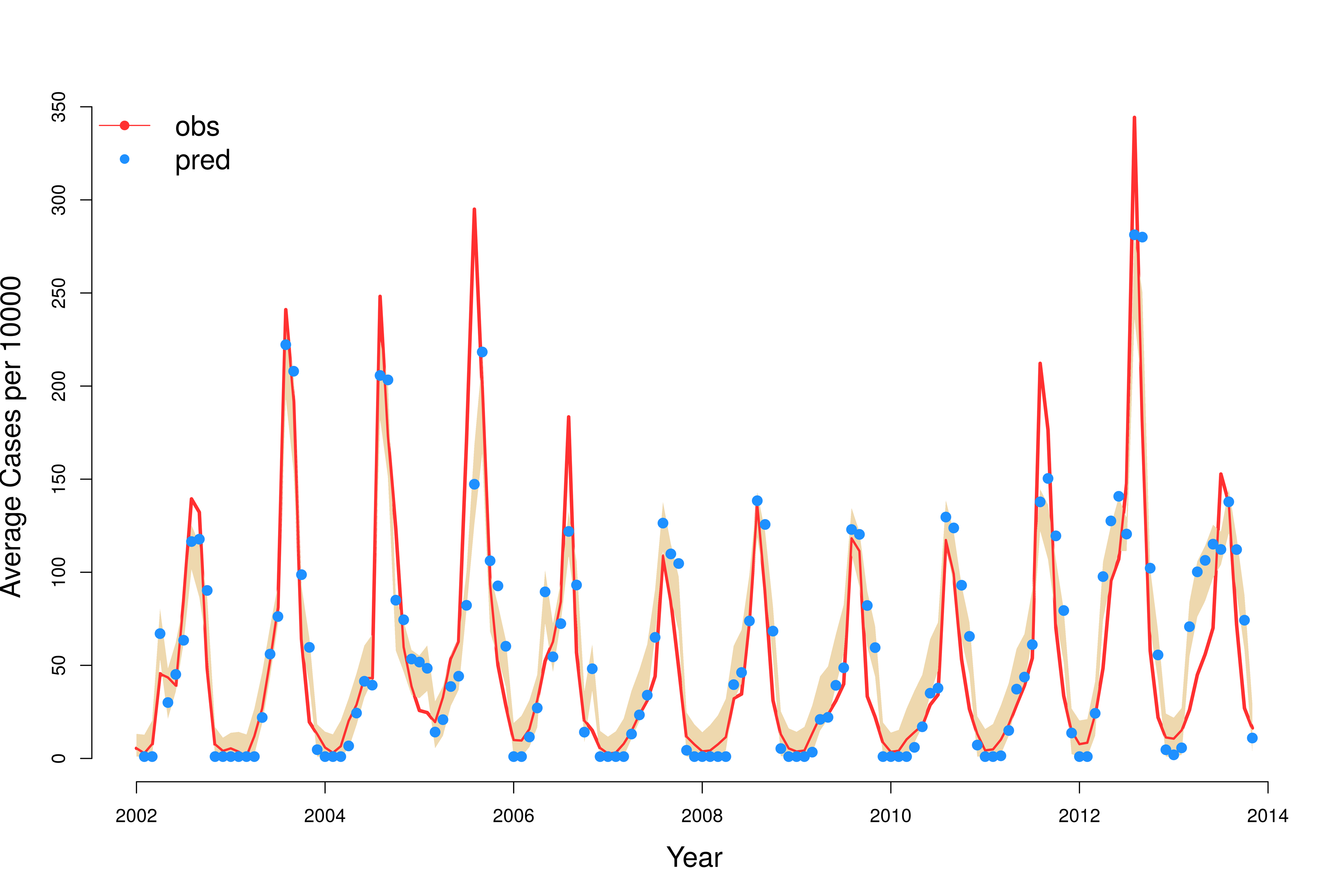

Supplement: S4 Fig — The red line corresponds to the average monthly cases per 10000 for the 59 wards. The blue dotted line corresponds to one-month ahead predictions for the median of the 5000 simulations values, and the light brown shaded region corresponds to the interval between the 5th and 95th percentiles for these simulations. (TIF) [file pntd.0005155.s004.tif]

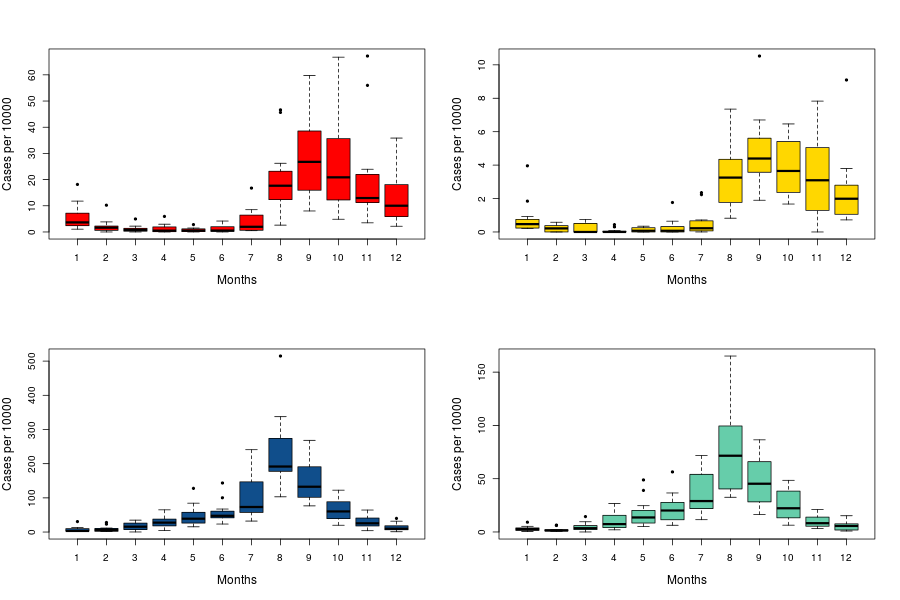

Supplement: S5 Fig — The top panels represent the seasonal pattern for Plasmodium falciparum, for the high risk region in the left and the low risk region in the right. The bottom panel shows the corresponding patterns for Plasmodium vivax. (TIF) [file pntd.0005155.s005.tif]

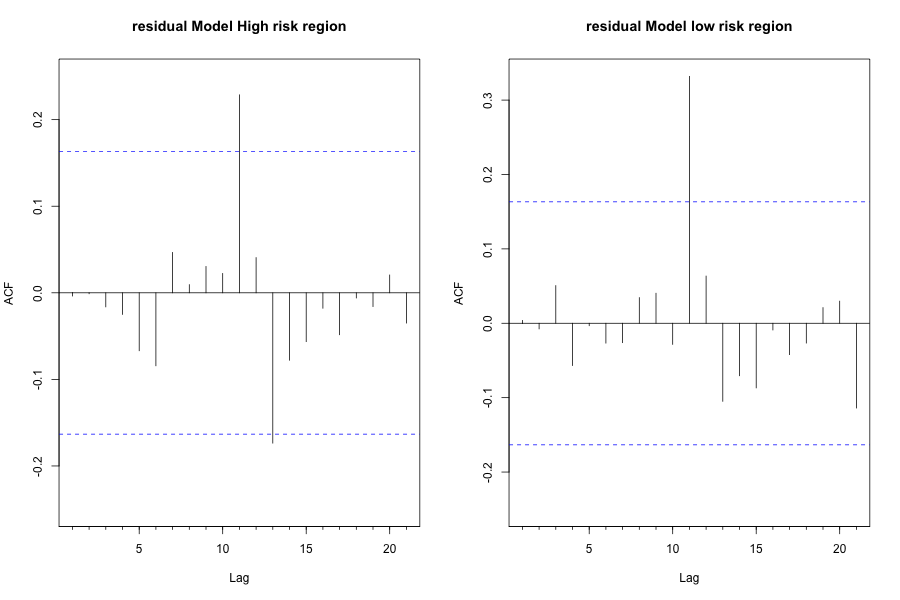

Supplement: S6 Fig — Although most of the autocorrelations fall within the confidence intervals, there is a small autocorrelation at lags of 11 and 12 months (seen in the significant spike of the ACF plot). This suggests that the model can be slightly improved by capturing the remaining seasonal variation. (TIF) [file pntd.0005155.s006.tif]

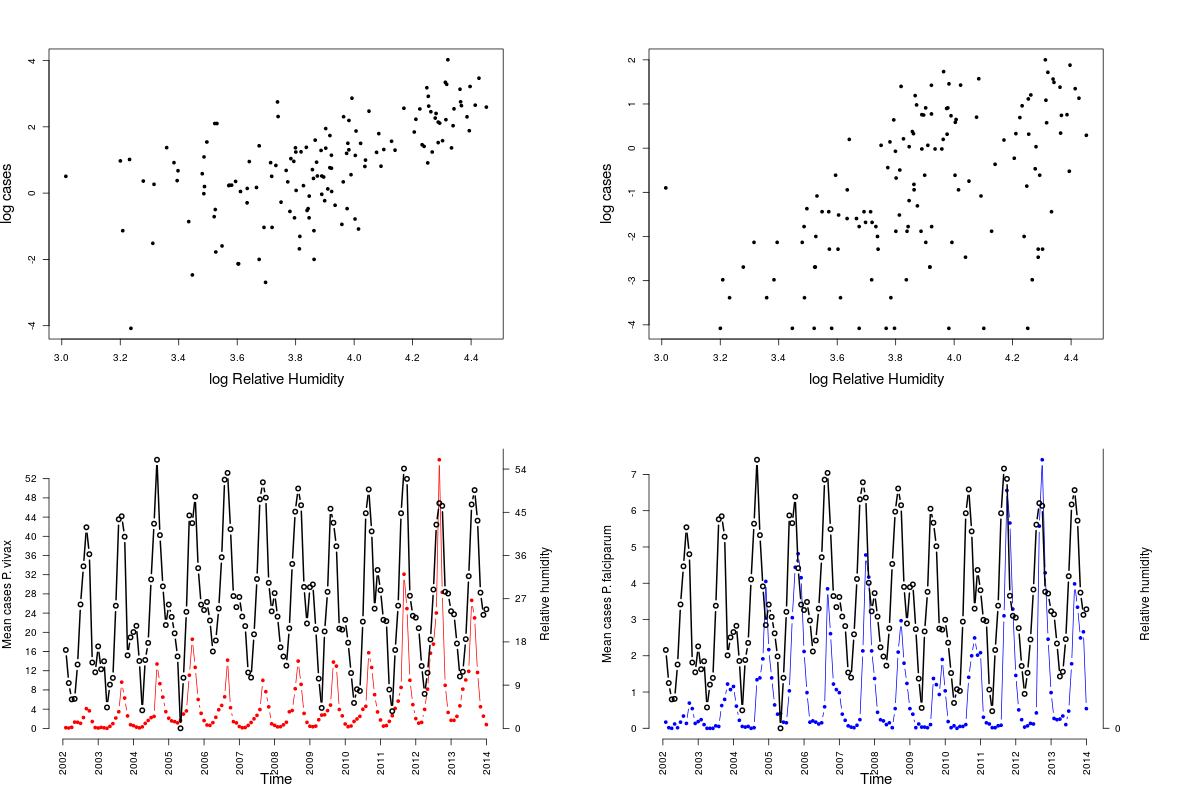

Supplement: S7 Fig — (TIF) [file pntd.0005155.s007.tif]

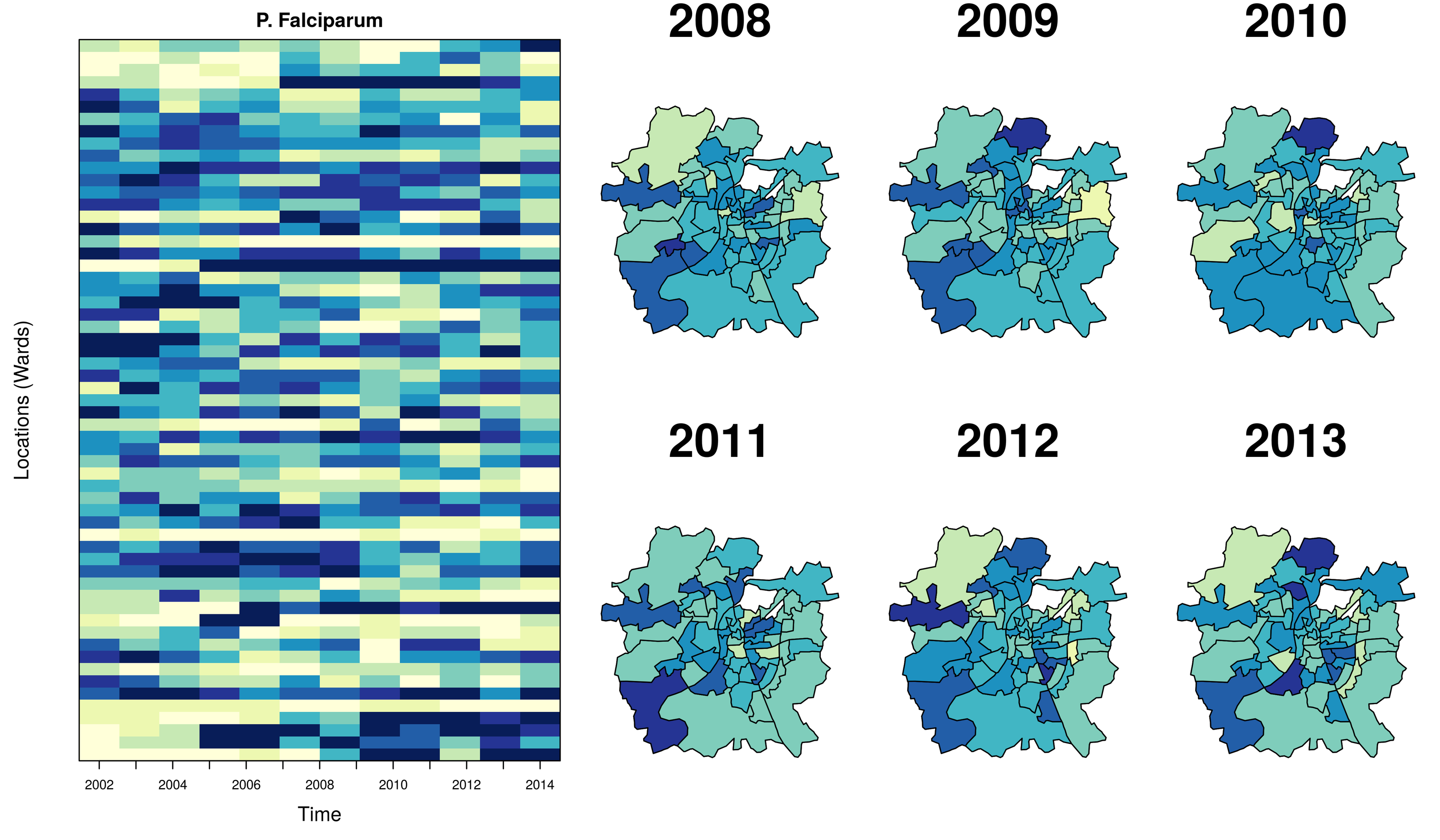

Supplement: S8 Fig — The panels show the distribution of the cases normalized by population, with the intensity of the color corresponding to the ranking of incidence. (TIF) [file pntd.0005155.s008.tif]

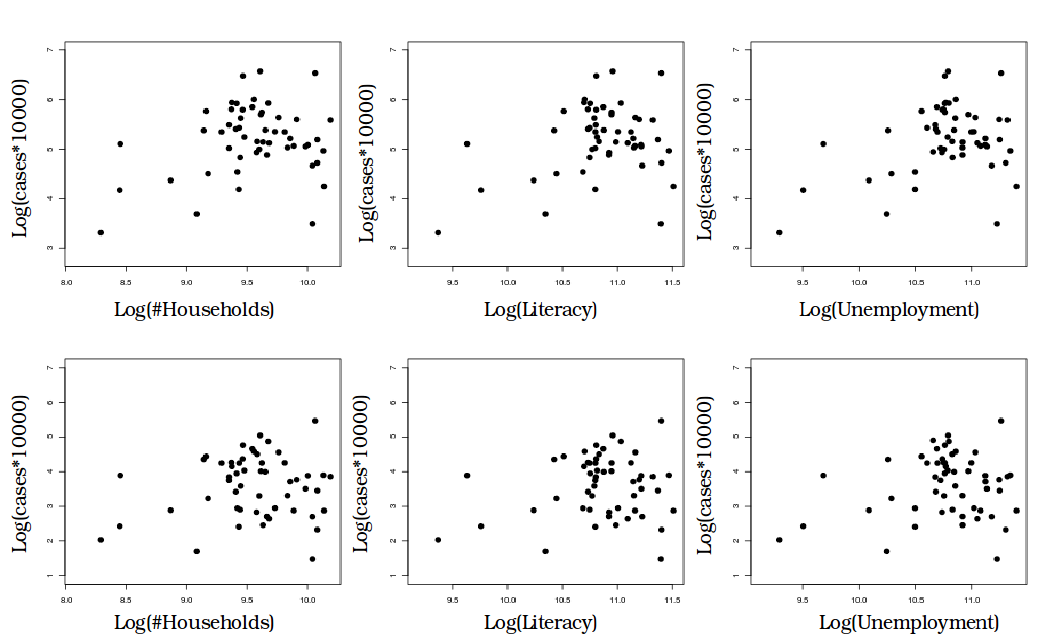

Supplement: S9 Fig — (TIF) [file pntd.0005155.s009.tif]

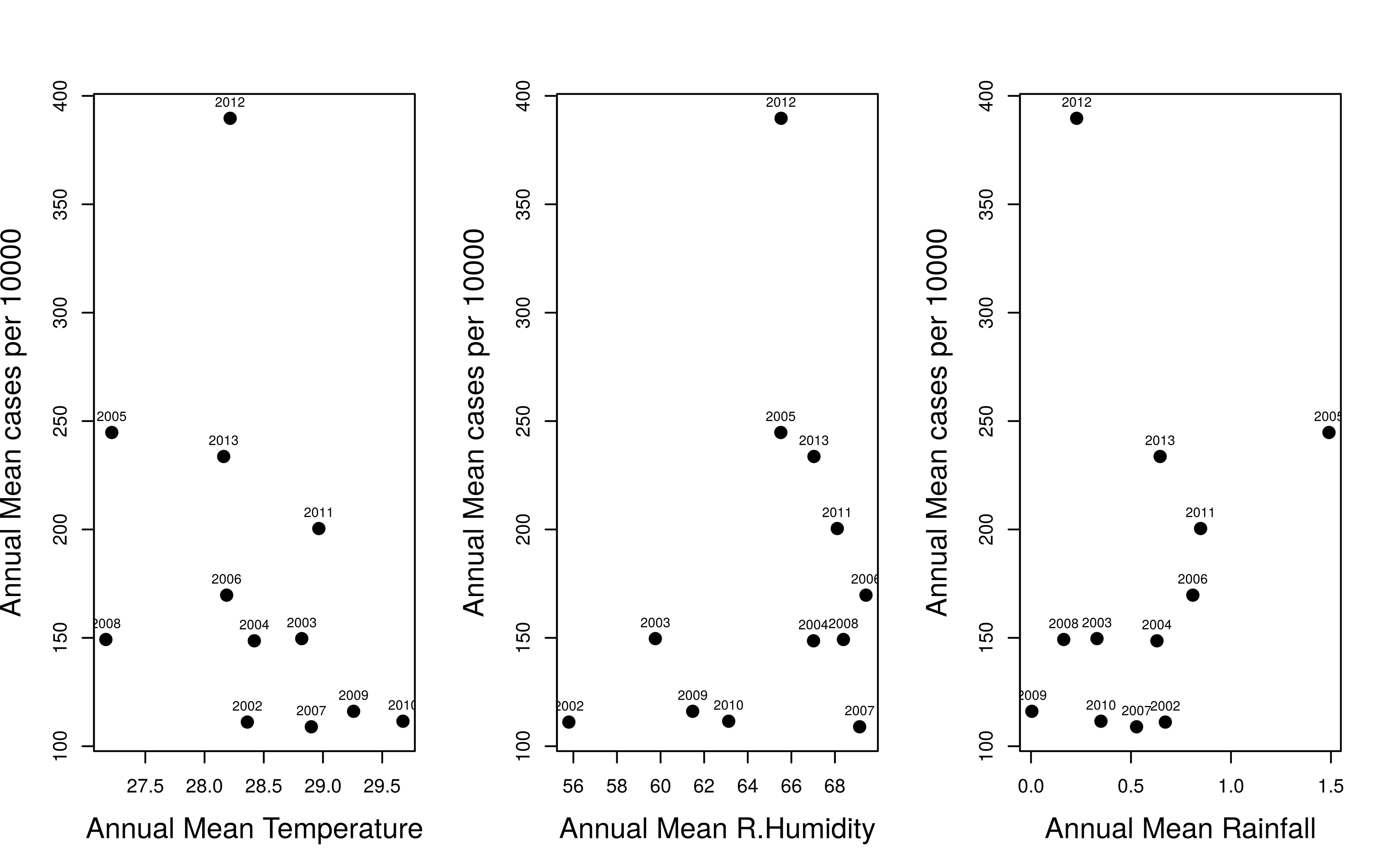

Supplement: S10 Fig — (TIF) [file pntd.0005155.s010.tif]

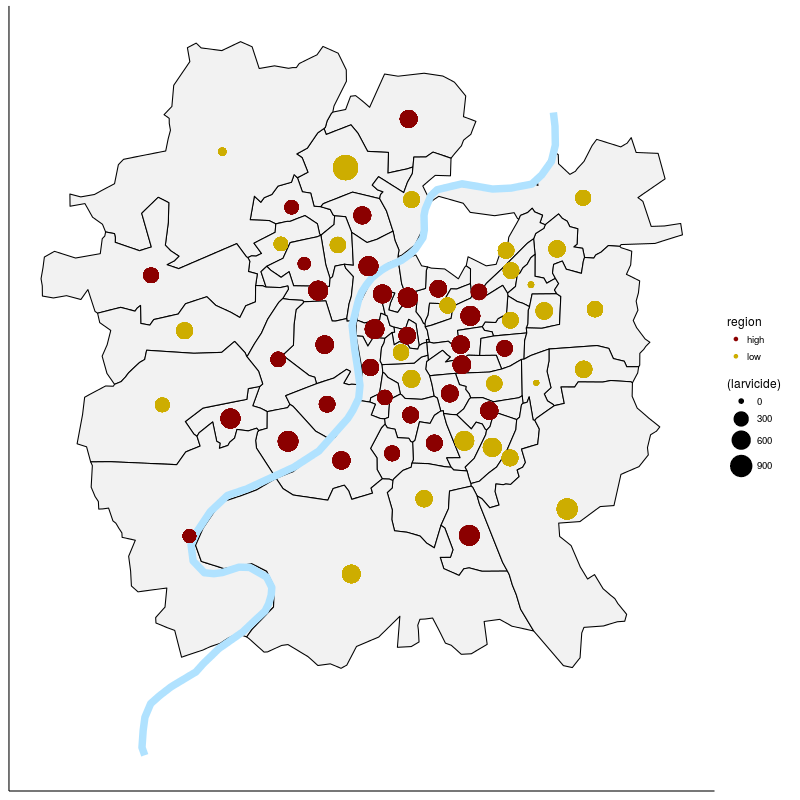

Supplement: S11 Fig — Red dots represent wards in the high risk regions, and yellow dots, those in the low risk region. (TIF) [file pntd.0005155.s011.tif]
