# Supplementary material for: Population Density, Climate Variables and Poverty Synergistically Structure Spatial Risk in Urban Malaria in India
Source: PLoS Negl Trop Dis. 2016 Dec 1;10(12):e0005155. doi: 10.1371/journal.pntd.0005155 (PMC5131912; doi:10.1371/journal.pntd.0005155)
Supplement: S1 Table — (DOCX) [file pntd.0005155.s012.docx]

**Table 1. Comparisons of nested models based on the likelihood ratio test (where asterisks indicate significance relative to the previous model) ).**

| **Model** | **HRR (High risk region)** | **LRR(low risk region)** |
| --- | --- | --- |
| **Full (autoregressive)** |  |  |
| **M1 (autoregressive + temperature) vs Full** | ******* | ******* |
| **M2(Autoregressive + temperature + rain) vs M1** |  |  |
| **M3 (Autoregressive + temperature + RH) vs M1** | ****** |  |
| **M4 (Autoregressive + temperature + RH + NDVI) vs M3** |  |  |
| **M5 (Autoregressive + temperature + RH + cases in the previous year) vs M3** |  |  |

***** significant at 95% ** significant at 99%**
