# Supplementary material for: Population Density, Climate Variables and Poverty Synergistically Structure Spatial Risk in Urban Malaria in India
Source: PLoS Negl Trop Dis. 2016 Dec 1;10(12):e0005155. doi: 10.1371/journal.pntd.0005155 (PMC5131912; doi:10.1371/journal.pntd.0005155)
Supplement: S2 Table — (DOCX) [file pntd.0005155.s013.docx]

**Table 2. Show comparisons of the zero inflated Poisson and negative binomial models fitted to the data.**

| **Test** | **Zero inflated** | **Negative binomial** |
| --- | --- | --- |
| **T test (likelihood ratio test)** |  | ******* |
| **AIC** | **749.1740** | **399.2133** |
| **BIC** | **785.5930** | **439.2742** |
| **Log lik** | **-364.5870** | **-188.6066** |
