# Supplementary material for: Population Density, Climate Variables and Poverty Synergistically Structure Spatial Risk in Urban Malaria in India
Source: PLoS Negl Trop Dis. 2016 Dec 1;10(12):e0005155. doi: 10.1371/journal.pntd.0005155 (PMC5131912; doi:10.1371/journal.pntd.0005155)
Supplement: S3 Table — The table shows the effects included in the model formulation and the corresponding total number of parameters. (DOCX) [file pntd.0005155.s014.docx]

**Table 3. Different parameterizations considered for the probabilistic model. The table shows the effects included in the model formulation and the corresponding total number of parameters.**

| Symbol | effect | Different effect for the two groups | Number of parameters (# groups) |
| --- | --- | --- | --- |
| P | Base | Yes | 18 |
| β | Neighbors | Yes | 6(2) |
| α | Seasonality | Yes | 6(2) |
| Se | Seasonality | Yes | 12(1) |
| Temp | Climate variability | Yes | 6 (2) |
| RH | Climate  variability | Yes | 6 (2) |
