# Supplementary material for: Population Density, Climate Variables and Poverty Synergistically Structure Spatial Risk in Urban Malaria in India
Source: PLoS Negl Trop Dis. 2016 Dec 1;10(12):e0005155. doi: 10.1371/journal.pntd.0005155 (PMC5131912; doi:10.1371/journal.pntd.0005155)
Supplement: S4 Table — (DOCX) [file pntd.0005155.s015.docx]

**Table 4.** **Model comparisons highlight the best model which incorporates the random effects, as well as the effect of temperature and humidity.**

| model | DIC |
| --- | --- |
| **Fixed effects model** |  |
| Temperature | 5645 |
| Temperature + Relative humidity | 5631 |
| Temperature + Relative humidity + rainfall | 5646 |
| **Fixed effect + Random effect** |  |
| Temperature | 5628 |
| Temperature + Relative humidity | 5622 |
| Temperature + rainfall + humidity | 5629 |
