# Supplementary material for: Population Density, Climate Variables and Poverty Synergistically Structure Spatial Risk in Urban Malaria in India
Source: PLoS Negl Trop Dis. 2016 Dec 1;10(12):e0005155. doi: 10.1371/journal.pntd.0005155 (PMC5131912; doi:10.1371/journal.pntd.0005155)
Supplement: S5 Table — Confidence intervals (CI) from posterior distributions (from two chains that are well mixed and have converged). (DOCX) [file pntd.0005155.s016.docx]

**Table 5.** **Estimated parameters for the best model which includes the effects of temperature and relative humidity, and the random effects, whose values are significantly different from zero. Confidence intervals (CI) from posterior distributions (from two chains that are well mixed and have converged).**

| **covariate** | **median** | **95% CI** |
| --- | --- | --- |
| **Temperature** | **0.1400** | **[0.117, 0.280]** |
| **Relative humidity** | **0.0532** | **[0.0245,0.0712]** |
| **Spatial structured parameter** | **0.2485** | **[0.1125,0.3578]** |
| **Overdispersion parameter** | **1.5195** | **[1.025,2.036]** |
