# Supplementary material for: Population Density, Climate Variables and Poverty Synergistically Structure Spatial Risk in Urban Malaria in India
Source: PLoS Negl Trop Dis. 2016 Dec 1;10(12):e0005155. doi: 10.1371/journal.pntd.0005155 (PMC5131912; doi:10.1371/journal.pntd.0005155)
Supplement: S6 Table — (DOCX) [file pntd.0005155.s017.docx]

**Table 6**. Statistical analysis of differences between the two regions identified using the socioeconomic information of the 2001 census for *P. falciparum*.

| *variable* | *statistic.t* | *parameter.df* | *p.value* | *log(mean of High risk)* | *log(mean of low risk)* |
| --- | --- | --- | --- | --- | --- |
| *Slum density* | 2.4701 | 47.6618 | 0.0171 | 3.3136 | 2.6065 |
| *Unemployment* | 3.0032 | 24.2349 | 0.0061 | 10.8310 | 8.5201 |
| *Marginal workers* | 2.8050 | 25.3346 | 0.0095 | 6.8985 | 5.4690 |
| *Literacy* | 2.3591 | 32.5098 | 0.0245 | 10.5786 | 8.5977 |
| *pop_below_6years* | 2.4111 | 32.5914 | 0.0217 | 8.8374 | 7.1430 |
| *Total population* | 2.3596 | 32.4772 | 0.0245 | 10.8835 | 8.8465 |
| *Area* | 1.5635 | 49.3197 | 0.1243 | 1.5391 | 1.2545 |
| *Number of Households* | 2.3569 | 32.5793 | 0.0246 | 9.3066 | 7.5628 |
| *Vulnerable communities* | 4.4633 | 29.4517 | 0.0001 | 6.3893 | 4.3684 |
| *Economically deprive communities* | 4.3176 | 26.7627 | 0.0002 | 8.8945 | 6.2612 |
