# Supplementary material for: Population Density, Climate Variables and Poverty Synergistically Structure Spatial Risk in Urban Malaria in India
Source: PLoS Negl Trop Dis. 2016 Dec 1;10(12):e0005155. doi: 10.1371/journal.pntd.0005155 (PMC5131912; doi:10.1371/journal.pntd.0005155)
Supplement: S7 Table — This model includes an autoregressive term that accounts for serial autocorrelation, the effect of relative humidity in both regions, and the effect of temperature in the low risk region. (DOCX) [file pntd.0005155.s018.docx]

**Table 7.** Results of the best negative binomial model selected by AIC for *Plasmodium falciparum* with climate covariates. This model includes an autoregressive term that accounts for serial autocorrelation, the effect of relative humidity in both regions, and the effect of temperature in the low risk region.

| Low risk region | | | | | | |
| --- | --- | --- | --- | --- | --- | --- |
|  | Estimate | Std. Error | z value | Pr(>\|z\|) | 2.50% | 97.50% |
| ar1 | 0.6295 | 0.0701 | 8.9850 | 0.0000 | 0.4658 | 0.7437 |
| intercept | -1.5647 | 1.4380 | -1.0881 | 0.2766 | -1.6098 | 0.2194 |
| temp | -0.0244 | 0.0446 | -0.5461 | 0.5850 | -0.0283 | 0.0271 |
| RH | 0.0297 | 0.0150 | 1.9855 | 0.0471 | -0.0023 | 0.0179 |
| High risk region | | | | | | |
|  | Estimate | Std. Error | z value | Pr(>\|z\|) | 2.50% | 97.50% |
| intercept | -0.695 | 0.467 | -1.490 | 0.136 | -1.610 | 0.219 |
| ar1 | 0.605 | 0.071 | 8.533 | 0.000 | 0.466 | 0.744 |
| RH | 0.037 | 0.016 | 2.354 | 0.019 | 0.006 | 0.068 |
