# Supplementary material for: Population Density, Climate Variables and Poverty Synergistically Structure Spatial Risk in Urban Malaria in India
Source: PLoS Negl Trop Dis. 2016 Dec 1;10(12):e0005155. doi: 10.1371/journal.pntd.0005155 (PMC5131912; doi:10.1371/journal.pntd.0005155)
Supplement: S8 Table — The best likelihood is obtained for the model that incorporates seasonality, temperature, two regions and the effect of neighbors. The last column shows the likelihood ratio test between the null model (model 1) and each of the other models. (DOCX) [file pntd.0005155.s019.docx]

**Table 8**. Likelihood comparison of the models showing the covariates included in each model. The best likelihood is obtained for the model that incorporates seasonality, temperature, two regions and the effect of neighbors. The last column shows the likelihood ratio test between the null model (model 1) and each of the other models.

| *models* | *seasonality* | *Neighbors* | *Temperature* | *RH* | *Regions* | *Log lik* | *DF* | *AIC* | *LRT* |
| --- | --- | --- | --- | --- | --- | --- | --- | --- | --- |
| *Model 1* | + |  |  |  |  | -5094.041 | 27 | 10242.08 |  |
| *Model 2* | + | + |  |  |  | -5073.091 | 33 | 10212.18 |  |
| *Model 3* | + | + | + |  |  | -5003.043 | 36 | 10078.09 | * |
| *Model 4* | + | + | + | + |  | -4986.389 | 39 | 10050.78 | * |
| *Model 5* | + | + | + | + | + | -3078.985 | 78 | 6313.971 | * |
| *Model 6* | + | + | + |  | + | -3272.393 | 72 | 6688.787 | * |
| *Model 7* | + | + |  |  | + | -4066.56 | 66 | 8265.121 | * |
| *Model 8* | + |  |  |  | + | -4615.592 | 54 | 9339.184 | * |
